# Exploring early- and mid-career academic work wellbeing challenges through a diversity and inclusion lens

**DOI:** 10.1186/s12909-024-05967-1

**Published:** 2024-09-27

**Authors:** Marianne Piano, Rebecca J. Jarden, Tandy Hastings-Ison, Belinda J. Lawford, Kristin Diemer, Flora Hui, Elaina Kefalianos, Gemma McKibbin

**Affiliations:** 1 Department of Optometry and Vision Sciences, Melbourne School of Health Sciences, University of Melbourne, Melbourne, Australia; 2 National Vision Research Institute, Australian College of Optometry, Melbourne, Australia; 3 Department of Nursing, Melbourne School of Health Sciences, University of Melbourne, Melbourne, Australia; 4 Austin Health, Melbourne, Australia; 5 Department of Physiotherapy, Melbourne School of Health Sciences, University of Melbourne, Melbourne, Australia; 6 Centre for Eye Research Australia, Department of Surgery, University of Melbourne, Royal Victorian Eye and Ear Hospital, Melbourne, Australia; 7 Department of Audiology and Speech Pathology, Melbourne School of Health Sciences, University of Melbourne, Melbourne, Australia; 8 Department of Social Work, Melbourne School of Health Sciences, University of Melbourne, Melbourne, Australia

**Keywords:** Academic, Early-career, Health sciences, Interprofessional, Professional, Tertiary education

## Abstract

**Background:**

Early- to mid-career academics (EMCAs) represent a core component of the Australian higher education workforce. These academics experience major challenges to their wellbeing, driving a strong desire to leave academia.

**Objectives:**

Determine (1) EMCA awareness of, and engagement with, previous University- and Faculty-level diversity and inclusion events/initiatives and (2) opportunities and solutions to address previously reported diversity and inclusion issues experienced in the workplace.

**Methods:**

114 EMCAs in medicine, dentistry and health sciences completed an electronic cross-sectional survey. The survey contained a list of University- and Faculty-provided diversity and inclusion initiatives and sought respondent ratings of interest, awareness (knowing/hearing about) and engagement (attending/applying/participating). Two in-person focus groups comprising participants who opted in during the survey or who responded to broader advertising were conducted. Both groups explored opportunities and solutions to address diversity and inclusion issues reported in an earlier organisation-wide survey.

**Results:**

Whilst early- and mid-career academics reported high interest in diversity and inclusion events, they also reported limited awareness and engagement with these events, feeling unsupported to engage or perceiving consequences for workload. Focus groups identified five themes related to opportunities and solutions to address diversity and inclusion issues experienced in the workplace (1) enhanced relational support for career progression, (2) clear and transparent processes for efficient working, (3) reducing structural barriers to create opportunity, (4) improved financial renumeration, and (5) improved transitions and pathways.

**Conclusion:**

Early- and mid-career academics often felt unable to engage with activities outside of their immediate work responsibilities, such as events about diversity and inclusion, due to feelings of high workload. A systems approach to deploying targeted strategies to address these wellbeing challenges is recommended to sustain and retain this critical workforce.

**Supplementary Information:**

The online version contains supplementary material available at 10.1186/s12909-024-05967-1.

## Introduction

Early- to mid-career academics (EMCAs) are central to the Australian higher education workforce; in 2021, 70% of academics were Level C (Senior Lecturer) and below [1]. These academics experience major challenges to their wellbeing, a long-standing problem [2] exacerbated by the COVID-19 pandemic [3], driving a strong desire to leave academia [4]. Academics directly influence the student experience, and the wellbeing of academics at work supports this influence to be positive [5]. Maintaining the wellbeing of EMCAs is critical. Recent data demonstrates that almost 40% of Australia’s researcher workforce is employed in the health and medical sciences. The academic-clinical nexus in these fields adds further complexity to academic roles without a clinical component, resulting in unique support needs for clinician researchers [4].

## Background

In a recent rapid review, workforce wellbeing of EMCAs was closely tied to diversity and inclusion concepts, linking through to the key recommendation for a carefully planned, global approach to development and evaluation of interventions that enhance wellbeing of early and mid-career academics [6]. Diversity can be split into two aspects of identity: social identity, such as being a parent, LGBTQIA + and disability; and professional identity, such as career stage, discipline and working hours [7]. Inclusion occurs when a diverse group of people are respected and connected and where they are progressing and contributing to organisational success [7]. One way of enhancing EMCA wellbeing could be through initiatives promoting diversity and inclusion, such as celebrating social identity, or funding opportunities to improve job security for those experiencing career interruptions.

Initiatives to promote diversity and inclusion are a major focus for universities, prompted by introduction of schemes such as the Athena Swan program [8], Rainbow Tick [9], and the Workplace Gender Equality Agency scorecard [10]. More recently, significant research funders are beginning to direct their attentions to university efforts in the area of diversity and inclusion, such as the Snow Medical initiative [11] and the NHMRC gender equity strategy [12]. It is evident that going forward, equity, diversity and inclusion will continue to be a priority area within academic institutions. However, it is well known that EMCAs grapple with unsustainable workloads in the face of significant job insecurity, experience power imbalances in the workplace, and do not always receive adequate supervision and mentoring [3]. These factors can all directly impact social and professional identity, as well as potentially affect ability to engage with interventions to enhance their wellbeing, such as diversity and inclusion events or initiatives. The level of engagement by EMCAs with diversity and inclusion events/initiatives is currently unknown, as previous surveys have focused on other important topics, such as mental health or job satisfaction.

### The local context

The current study builds from a previous local university faculty-wide survey focusing on wellbeing and workplace culture for early and mid-career researchers [3]. In this previous survey, key findings included unsustainable workloads, inadequate supervision and mentoring, job insecurity, and burnout. Subsequent university and faculty-level diversity and inclusion strategies have failed to take into account these key wellbeing challenges faced by the EMCA workforce. We therefore undertook this mixed methods study to explore early- and mid-career academic work wellbeing challenges through a diversity and inclusion lens. This comprises two facets: (1) EMCA awareness of (knowing/hearing about) and engagement with (attending/participating) faculty and university-level diversity and inclusion events/initiatives, and (2) a qualitative exploration of the key issues identified in the previous survey [3].

### Research questions

For early and mid-career academics working in a Medicine, Dentistry, and Health Sciences Faculty within a university:

RQ1: What is awareness of, and engagement with, previous University- and Faculty-level diversity and inclusion initiatives?

RQ2: What opportunities/solutions exist to address previously reported wellbeing issues experienced in the workplace?

## Methods

### Ethical approvals

The research protocol was approved by the [masked] Human Research Ethics Committee (2022–22643-25,636–3). The research methods including recruitment, data storage and confidentiality were conducted in accordance with ethical guidelines and regulations. Informed consent was obtained from all participants in this study.

### Study design, setting and participants

Adopting a linear sequential mixed methods study design, our participants were early and mid-career academics working in a health faculty at a large metropolitan university. This Faculty comprised health sciences, population and global health, medical, dental, psychological sciences and biomedical sciences, as well as affiliated medical research institutions. This was a single-site study as the research project originated from an internal faculty funding application by the research team. Early and mid-career academics were defined as university staff of academic pay Levels A-D within the Australian system, translating to anyone of Assistant Professor rank or below. The exact number of early and mid-career academics in this Faculty could not be confirmed, but in 2021, there were 1465 academic staff in the Faculty. Using Department of Employment, Engagement and Workplace Relations institution-level statistics, which estimate 60% of all institutional staff are levels A-C, 879 staff could be early- or mid-career. It was not possible to separate data for Level D staff, therefore these estimates are conservative.

Designation as an academic encompasses teaching-focused, research-focused, teaching- and research-focused and academic specialist posts. Academic specialists are postdoctoral staff members engaged in specific roles, such as research data analysts. Professional staff and postgraduate research students studying for a PhD were excluded from the sample, but academic staff undertaking a PhD alongside their teaching contract were included. This ensured a focus on academic members of staff, as there is a substantial body of research focusing on tertiary education student experiences and perceptions of diversity and inclusion. We chose to include teaching-only academics as well as researchers, as recent surveys in this area have focused purely on early and mid-career researchers [4].

We conducted an online cross-sectional survey on the Qualtrics platform from March to May 2022, to determine awareness of, and engagement with, diversity and inclusion initiatives. Subsequently, we held two in-person focus groups at the University, in May 2022. These focus groups explored, from a diversity and inclusion perspective, key wellbeing challenges experienced by EMCAs in the workplace and opportunities/solutions to address them. These challenges were identified via surveys undertaken by this same faculty and one other University, prior to commencement of this study [3].

### Recruitment procedures and sample size

The survey was primarily advertised through University-wide and Faculty-wide staff newsletters and repeated email communications from the Faculty diversity and inclusion team, as well as on social media accounts belonging to the Faculty media team, diversity and inclusion team, and members of the research team. Participants either opted into the focus groups when responding to the cross-sectional survey, or through responding to separate advertisements via the channels stated above.

Although no statistical analyses were planned for the cross-sectional survey to necessitate a target sample size, we aimed for a minimum of 100 respondents. A previous survey targeting the same population within our institution had an estimated 17% response rate (*n* = 150). Assuming a population of 950 ECMAs (consistent with the approximate number of EMCAs within our Faculty), a minimum sample of 100 people would ensure a maximum width of 9% in the 95% confidence intervals around the response proportions. For the focus groups, we aimed to have between 6–8 people per group, to ensure equal opportunities to contribute within the 2 h session. We had originally intended to conduct four focus groups of this size, but difficulties with recruitment and scheduling meant that only two focus groups could take place.

### Survey design, conduct and analysis

The survey exploring engagement with diversity and inclusion event/initiatives was developed by members of the research team from reviewing University event websites and email communications received. The survey was then peer reviewed by the research team who were of multiple EMCA disciplines included in the final survey, to ensure the phrasing of the questions made sense, that the demographic information collected was relevant, and that the survey as a whole met the stated aims of this part of the project. The survey comprised 26 items, reported in full in Supplementary File 1. Demographic information such as age and details about social identity (e.g. caring responsibilities, gender, disability status) and professional identity (e.g. academic level, contract type, holding of clinical role) were collected. For University and Faculty-level diversity and inclusion events/initiatives held from March 2021 to February 2022, respondents were invited to indicate, for each, their level of:

1. Awareness (whether they recalled hearing about it or seeing it advertised)
2. Interest (whether they would have liked to engage with it)
3. Engagement (whether they attended or participated)

A free text box was provided for respondents to add any further comments regarding University or Faculty-level diversity and inclusion events/initiatives. Respondents were also able to indicate interest in future focus group participation regarding these topics.

The survey data was cleaned by excluding incomplete entries (e.g. where demographic information had been completed but no other parameters) and flagged entries associated with automated form fillers (survey bot) completions as noted by Qualtrics. Free text underwent content analysis and descriptive statistics were reported for other variables.

### Focus group procedures and analysis

The topic guide for the two focus groups (Supplementary File 2) was informed by a previous Faculty-wide survey focusing on wellbeing and workplace culture for early and mid-career researchers (Singh et al., 2020). Each 2-h focus group was audio-recorded and transcribed. Transcripts were checked by facilitators of the focus groups (MC, GM), transcripts were then uploaded to NVivo™ (qualitative data software; QRS International, Victoria AU). Transcripts were analysed using the inductive thematic analysis approach [13]. This method was selected as it can accommodate a collaborative approach. This project represented an opportunity for other members of the research team to gain experience in qualitative data collection and analysis methodologies under supervision of an experienced researcher (GM). Members of the research team read the transcripts to develop initial thematic codes (GM, MC, TH, FH, RJ). These codes were then reviewed and refined before developing and finally naming themes (GM, MC, RJ). The narrative summary of themes was then constructed by the same team in the context of the Diversity Council of Australia definitions for social identity, professional identity and inclusion.

### Reflexivity and trustworthiness

As a research team, we adopted a reflexive stance, discussing preconceived expectations related to the research and how these expectations influenced our research process; from developing the research questions through to analysis and reporting [13]. Reflexivity enhanced our ethical and political self-awareness, both of which were embedded in our professional and academic roles as female, early/mid-career academics, working in the health sciences school of a tertiary educational organisation. Our process of interprofessional peer-review and debriefing supported our conceptualisation [14]. During this process we explored our positioning and influence on the research. Findings are reported as a narrative summary and tabulated questions, themes, and participant quotes. Themes were developed from a range of quotes from participants.

## Results

### RQ1: What is your awareness of, and engagement with, previous University- and Faculty-level diversity and inclusion initiatives?

114 EMCA respondents (defined as Levels A-D) from across the Faculty’s many Schools and affiliated research institutes completed the survey. Mean age was 41.4 ± 8.7 years (missing *n* = 4), and respondents had worked within the faculty for between 6 months and 30 years, experiencing a median of 3 contract renewals (range 0–25, IQR = 5, missing *n* = 6). Sample characteristics in relation to social identity are shown in Table 1. The majority of respondents were women (*n* = 99, 87%) and many in the sample were parents of school age or younger children (*n* = 58, 51%).

**Table 1:** Social identity characteristics of respondents (*n* = 114)

Characteristic	*n* (%)
Parent of school-age or younger children	58 (51%)
Identify as person of colour	22 (19%)
Caring responsibilities other than parenting	21 (18%)
Identify as LGBTQIA +	15 (13%)
Neurodiverse	11 (10%)
Living with a disability	7 (6%)
Strongly adhere to a religious faith	4 (4%)
Identify as a woman	99 (87%)

**Fig. 1:**
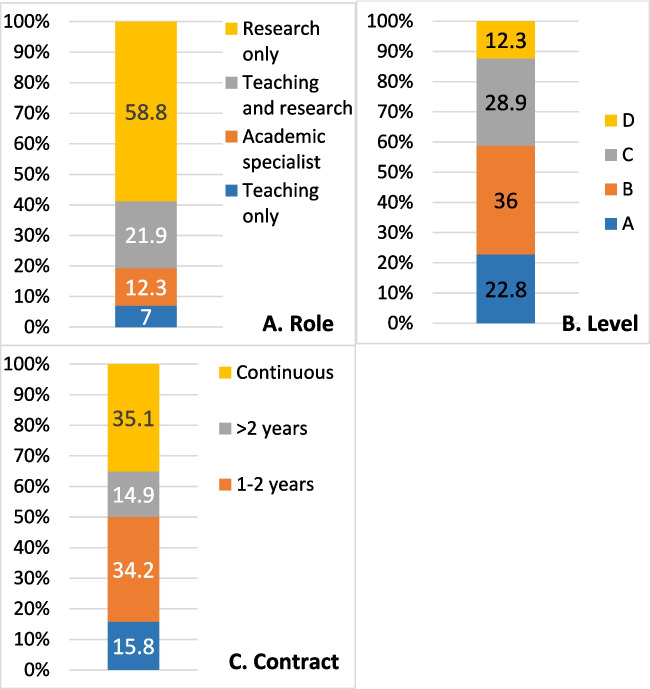
Professional identity characteristics of the respondents (*n* = 114)

Sample characteristics in relation to professional identity are shown for role (Fig. 1A), academic level (Fig. 1B) and contract (Fig. 1C). Regarding role, 47% (*n* = 54) worked part time, and 17% held a clinical role (*n* = 19). A range of contract lengths were represented in the sample; from casual to continuing. Largest proportions were on either a continuing (35%) or fixed term, 1–2-year contract (34%). The most prevalent academic level was B (36%), although Levels A-D are represented (12% Level D). Within the sample as a whole, 61% (*n* = 70) had held external funding, with the majority of this group (*n* = 58) holding such funding as a principal investigator. Holding of internal funding was similar (*n* = 59, 52%, *n* = 50 as principal investigator). Of the group, 75% (*n* = 85) already held a PhD, and 47% (*n* = 53) had been invited to apply for promotion.

Whilst there was a range of interest levels in diversity and inclusion events, there was moderate awareness, and low levels of engagement at faculty (Table 2) and university levels (Table 3). Mean interest was 45% ± 20% at Faculty level, ranging from 2–76%, and 51% ± 13% at University level, ranging from 32–84%. Mean awareness was 42% ± 27% at Faculty level, ranging from 6–90%, and 26% ± 19% at University level, ranging from 7–68%. Mean engagement was 13% ± 14% at Faculty level, ranging from 1–54%, and 10% ± 11% at University level, ranging from 0–35%. Interest, awareness, and engagement were higher for nationally recognized in-person events such as Reconciliation week (70.5%, 68% and 26%, respectively) and IDAHOBIT (47%, 41% and 16.5%, respectively). Events communicated via a single e-mail or local news notification were less known. The most popular internal faculty event was the Supporting Women in MDHS Faculty (SWiM) seminar series, in which a variety of women role models at different career stages are interviewed, exploring the journey to leadership. This is advertised by email on a regular basis and has a dedicated web-page where past seminar recordings can be viewed. Awareness, interest and engagement in this event was 90%, 76% and 54% respectively.

**Table 2:**
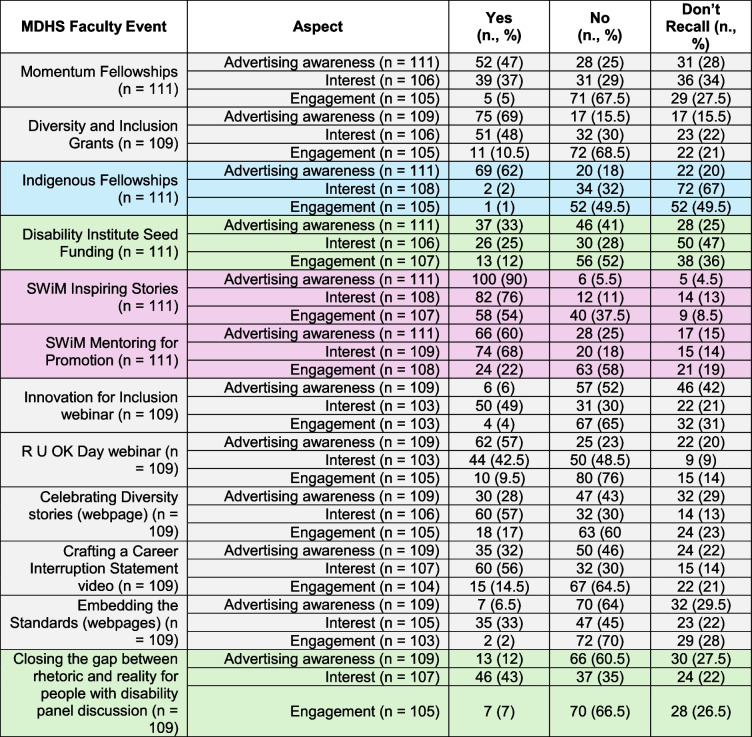
Interest in, advertising awareness of, and engagement with Faculty level events

Colour key: Indigenous, Gender, LGBTQIA+, Disability, Other

**Table 3:**
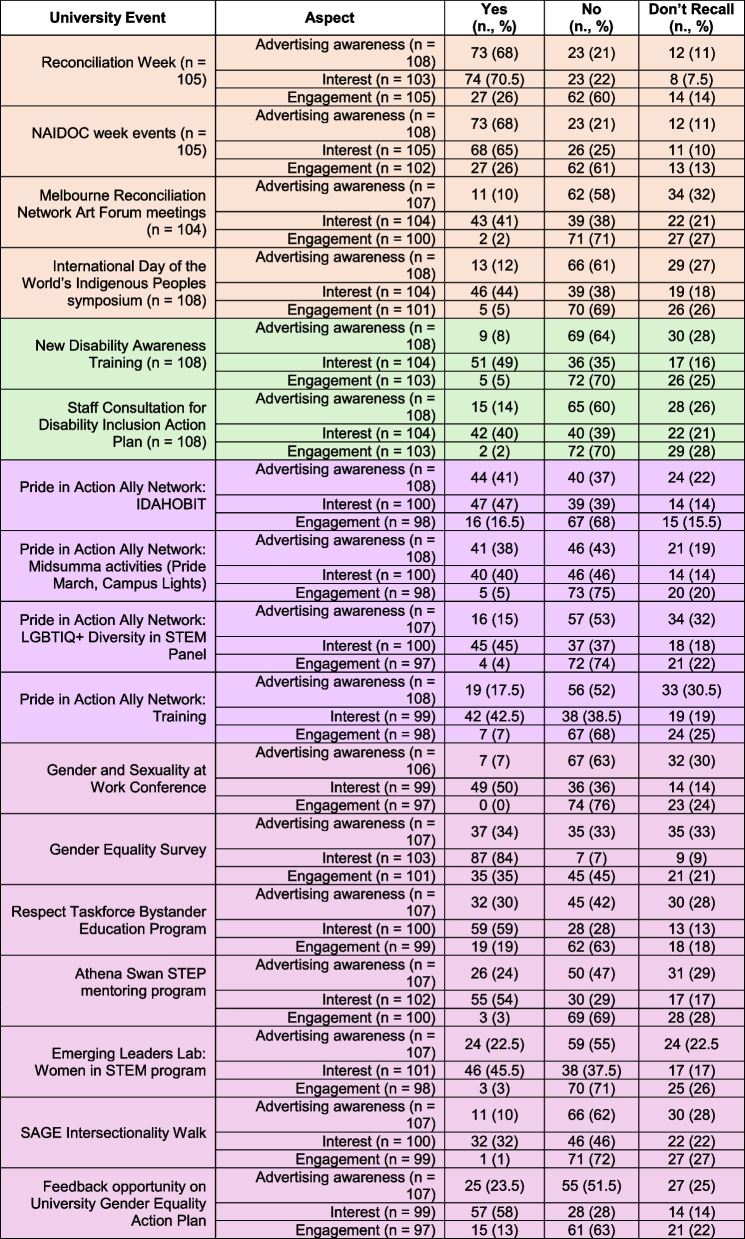
Interest in, advertising awareness of, and engagement with University level events

Content analysis of free text responses is shown in Table 4. This indicated early- and mid-career academics feel extremely time-poor (*n* = 13), which limited their ability engage with events and initiatives outside their immediate workload. There was a perceived lack of support from their immediate line management to engage with diversity and inclusion events/initiatives (*n* = 3). Some implied consequences of engagement such as having to make up the time, resulting in selective engagement with opportunities considered to be of sufficient relevance/value. Social and professional identity aspects such as part-time working, being located off the main campus and caring responsibilities were also barriers to engagement (*n* = 5).

**Table 4:** Content analysis of free text responses (*n* = 56)

Topic	Count
Activities outside main campus limited	2
Number/range of events is great/impressive	2
Too many events	5
Some events are not very diverse or feel like window-dressing, rather than addressing needs of the groups celebrated, or leave some groups unrepresented e.g. men of colour	3
Not clear whether time and resources spent on diversity and inclusion initiatives are achieving change	4
Often feel these are not relevant to me or unsure if I am eligible without having to hunt for more information	7
I think I miss information about these or don't remember them being advertised	5
I am too busy with work commitments to attend, or already engage in many hours of unpaid work	13
I am interested in these events or would like to be more engaged	5
I like to hear about these events even if I can't go	4
I was unable to attend due to part time working, caring or parental responsibilities/leave	3
I feel unsupported by line management to meaningfully engage with events/initiatives during my work hours, or I must catch up on work missed in my own time	3

EMCAs also indicated they are not aware of some diversity and inclusion events (*n* = 5), or felt they received too many emails, with some using email inbox categorisation strategies to manage email volume and prioritise workload, such as filtering out faculty and staff newsletters. Some EMCAs felt overwhelmed by the number of events (*n* = 5), although others felt it was better to have a range of events than no action (*n* = 2). Information about events and initiatives could be made clearer to help EMCAs decide about their relevance in relation to their professional or social identity, and whether to engage (*n* = 7). Lastly, there is some distrust, with diversity and inclusion events/initiatives seen as window-dressing rather than addressing needs (*n* = 3), or achieving change (*n* = 4). Despite these issues, EMCAs indicated that they were interested in many of the advertised events and liked to hear about them even if they were unable to attend (*n* = 9).

To summarise, the survey indicated that EMCAs were interested in diversity and inclusion events and initiatives, but key challenges included low awareness due to how they were advertised (mainly by email), feeling unsupported to engage, not having the information to decide about relevance/eligibility, and high workloads or aspects of social and professional identity limiting time/capacity to engage. Events tied to national or global initiatives such as Reconciliation week and IDAHOBIT (International Day Against Homophobia, Transphobia and Biphobia), or frequently advertised by direct emails rather than generalised newsletters (e.g. SWiM) had higher proportions of both awareness and engagement.

### RQ2: What are the opportunities and solutions to previously reported wellbeing issues experienced in the workplace?

In total, across the two focus groups, 10 EMCAs participated, with the majority being Levels B and C. All were women. Only one Level D and one Level A participated. Age range was 29 to 61 years. Time spent working at the University varied from 2 to 30 years. We constructed five themes through our analysis of the focus group data: (1) Enhanced relational support for career progression, (2) Clear and transparent processes for efficient working, (3) Reducing structural barriers to create opportunity, (4) Improved financial renumeration, and (5) Improved transitions and pathways.

#### Theme 1: Enhanced relational support for career progression

EMCAs identified initiatives that could help address the issue of inadequate supervision and mentoring, and job insecurity. With regard to professional identity, support from senior staff to enable career progression for academics within teams was an important topic, with solutions including better guidance and incentives for supervisors to adopt a supportive role, along with increased accountability, supported by appropriate training. Further, EMCAs wanted mentoring from a variety of sources and realistic role modelling of work-life balance from senior staff, particularly women, speaking to social identity.

Within this theme there was an emphasis on accountability for senior staff to demonstrate responsibility for supporting career development of their supervisees, including around job security issues and publications:

*I actually think that there needs to be built into the performance of those more senior academics some accountability around, well, what is – let's have a look at your contracts. It looks like you have a lot of casual contracts. You seem to have a lot of fixed-term contracts. (Focus Group 2, P006)*

*It's about also looking at the authorship. Okay, so you've had all these people on casual contracts. Let's have a look at your publications. I'm talking about senior – let's have a look at where are the – where are [the junior staff] reflected in these publications or in these outputs? Where is the recognition of the work that they've done? (Focus Group 2, P006)*

However, EMCAs recognised the need for senior staff to have more support and training in how to manage staff teams:

*There’s obviously training that you can do to in terms of supervising students, but there’s not much training in terms of management supervision and supervising teams and different things. I think there’s - we can better equip our supervisors and managers to – with the right skills to do their job*. *(Focus Group 1, P004)*

Senior academics needed particular training in how to manage issues associated with job insecurity such as staff contracts, especially when project start and finish dates did not match up with contracts:

*More support to senior staff [is needed] to be future planning around contract project dates, particularly where those stopgaps come up and where staff are working on multiple projects. So I have a full-time position, but I'm across four different projects, I'm funded by four different projects. Those deadlines and/or project end dates don't always match up, so you have a point where you kind of go, okay, well, there's only funding for half of my time, whereas I'm in a full-time position, so where does that come from? (Focus Group 2, P007)*

EMCAs suggested that mentorships do not have to reflect the academic hierarchy and could reflect a peer-to-peer support model:

*Mentoring doesn’t have to be from senior staff, necessarily. I feel like there’s room for peer support and mentoring, and not necessarily from people with the same years post-PhD. I guess I want to try and get away from that whole hierarchical framework, and almost – I don’t know if this is possible but finding people in the same boat as you. (Focus Group 1, P002)*

Visible role modelling of a healthy approach to work-life balance was also considered important by EMCAs, who wanted to see more women leaders demonstrating realistic working situations, but also raised questions about whether a healthy work-life balance was achievable in the face of overwhelming workloads:

*How could [female leaders role modelling healthy work-life balance] actually do the job in the time that they’re paid to do it anyway? (Focus Group 1, P003)*

Overall, EMCAs wanted senior academics to be more accountable to the career progression of junior staff and to have better training in how to manage staff contracts to afford greater job security.

#### Theme 2: Clear and transparent processes for efficient working

EMCAs identified strategies to make their workloads more manageable to avoid burnout. These strategies included transparency around working conditions and the realities of academia as part of the onboarding process, reflecting a key aspect of professional identity, as well as clear policies relating to flexible working arrangements, cover for leave, and time in lieu, vital to accommodating facets of social identity such as being a carer or having dependants. They suggested that more thorough onboarding and induction processes would facilitate efficient work practices:

*There’s a lot of inconsistency in induction to university systems and tools and the way things work in the University. So, I think there’s room to do some better onboarding and system induction processes, because I know a lot of people try to do something and don’t realise that there’s already an existing University system, or a – or somebody else doing this work. (Focus Group 1, P004)*

Induction should also include a transparent conversation about work conditions at the University, including the long working hours and short-term contracts:

*I really do think when people are coming into academia, whatever level, but particularly the A and B, people actually need to know what they're coming into. What these contracts mean, what they can mean down the track in terms of being continually placed on casual contracts, fixed-term contracts*. *(Focus Group 2, P006)*

EMCAs discussed working more than their contracted hours, and during annual leave, with the associated confusion about how to take time in lieu or gain reimbursement for work completed:

*Time in lieu was really tricky. Like how would you formalise that? For instance, I just took 10 days off between Easter and ANZAC day, during which time I wrote two grant applications and did a poster for a conference*. *(Focus Group 1, P003)*

*Thinking about taking time in lieu. I think some teams probably do that in more informal ways – I think if you want to make it available as something that people see as supporting their flexible workload, then I think it needs to be internally monitored somehow, whether there’s a system that counts hours and pays back time or [some other system]. (Focus Group 1, P004)*

Flexible working arrangements were considered important to support EMCAs with caring duties:

*Different time fractions and flexible work arrangements; I think they're critical, because if your child's got an activity on and it's important for you to be there, then I think they should be able to go there and see it, because that child's only going to be that age at that time and that moment in time is never coming back*. *(Focus Group 2, P008)*

*I know some people prefer to keep their time very set and stable, but others don’t. There probably needs to be a process of, I guess letting people – giving them the option of opting in or opting out of different flexible working opportunities. Maybe that’s a discussion between supervisors and their teams*. *(Focus Group 1, P004)*

The value of professional staff was highlighted and discussed as another strategy to enhance working efficiency. EMCAs acknowledged the limited capacity of professional staff to assist with these processes due to University reductions within this workforce:

*The various different rounds of getting rid of people that actually were helpful has meant we have to do – we do all of our HR, we do all of our environmental health and safety. Even the ordering. My research assistant used to do it. Now I don't have one, so everything is just hugely time-consuming. All the short-term, small amounts of funding means a new research contract which has to go backwards and forwards. (Focus Group 2, P006)*

It was also noted that professional staff were disproportionately allocated to supporting senior staff:

*I really do think it's about increasing the professional support roles. They're fantastic in terms of the support they provide, but I feel like it's skewed very much to the more senior academic staff*. *(Focus Group 2, P006)*

EMCAs identified the main sources of burnout to be tight timelines for project deliverables and absent backfill positions during leave:

*The lulls between highly pressured deliverable periods where if you're on multiple projects [could guard against burnout]. You've got multiple competing deadlines and there's never sort of a breathing space; so trying to find those spaces*. *(Focus Group 2, P010)*

*Thinking about the burnout in terms of when you're on leave, the work just waits for you until you get back. So just thinking about if there's projects or a project team's thinking of – you know, for project leads or the team themselves to be really thinking about, well, who can pick up this work? Who's got the skillset? I don't know if there's enough of that, those conversations, that occur for people to actually pick up each other's work. (Focus Group 2, P007)*

EMCAs felt that regular conversations with supervisors about workload would be helpful to reduce burnout:

*Whether something like that could be built into projects or between – you know, with your direct manager. It doesn't have to be fortnightly, but monthly where you're just doing a bit of a check in to see how the person's travelling with their workload. Because that's when you can have those conversations about workload and capacity. (Focus Group 2, P007)*

#### Theme 3: Reducing structural barriers to create opportunity

EMCAs reflected on opportunities to address issues of burnout and unsustainable workloads related to equalising structural disadvantage associated with social identity; primarily gender and mental health issues. They recommended that caring commitments be recognised and normalised, and that gendered workplace disparity is corrected. In addition, EMCAs stressed the importance of accessible mental health support and leave, when necessary.

In terms of caring commitments, EMCAs identified that women often carry the load of caring responsibilities in the family, and that this is not always recognised or valued in the workplace:

*I think that's critical that it's not [colleagues saying], “Oh God, there they go again watching their children perform” - that it's actually part of the [workplace] culture that we should actually celebrate and accept it as a normal. (Focus Group 2, P008)*

It was also noted that women tend to pick up the extracurricular activities/responsibilities in the workplace, increasing their workload, and that this is not necessarily acknowledged. Women were carrying voluntary extracurricular activities at work, as well as the majority of caring responsibilities at home:

*[I would like] acknowledgement that particularly for women, you're carrying the load of family as well . . . [There should be] some sort of attention to the fact that you did pick up those extra things [at work] that often men don't – that [men are] not carrying that. (Focus Group 2, P006)*

EMCAs wanted the unpaid labour picked up by women in the workplace, to be made visible and talked about:

*[Managers should lead] more open discussions around the informal extra duties that come up if you're – particularly around team or community building, extra events, that sort of thing, that often the women in the team will take up and it's not funded, it's not paid*. *(Focus Group 2, P010)*

Participants also commented on the complete lack of gender diversity within the focus groups, which were made up exclusively of female EMCAs. They suggested that this lack of gender diversity in the research sample reflects how women tend to take on unpaid workplace duties that contribute to bettering workplace culture:

*I just hope that there’s a bit more of a gender balance in the other focus groups. I’m noticing that we’re all talking about our ridiculous workloads and all the extra-curricular stuff that we do, but it is often female academics who are volunteering for these sorts of things to improve the workplace culture, and we can’t do that all on our own, because we all benefit if things get better. (Focus Group 1, P003)*

In addition to issues relating to addressing gender inequity and its impact on workload, EMCAs drew attention to the need for academics experiencing mental health issues, including burnout due to unsustainable workloads, and to be able to take “mental health days” rather than sick leave or annual leave:

*It needs to be acknowledged that it's not using an annual day [for mental health related issues]. You shouldn't have to run off to a doctor to get a certificate either. It's – because then it's – if you do it then you've defeated the purpose, because you've had to book into a doctor and had to pay for that, so it defeats the purpose of this day of pressure release*. *(Focus Group 2, P008)*

Further, it was thought that mental health support should be free and accessible to EMCAs:

*I think it should be a free service that's provided and available at the University, because, again, some other corporations or companies or businesses have free mental health services within. It needs to be easy to get. If I'm having a crisis, there's no point in getting an appointment in three months' time because that's when I get a book in, because the crisis is now and that's the issue. (Focus Group 2, P008)*

Overall, EMCAs identified structural gender inequality impacting workload and burnout. They wanted this disparity acknowledged and addressed. Further, they drew attention to the need for more accessible mental health leave and on-campus mental health support services.

#### Theme 4: Improved financial renumeration

EMCAs reported that workload sustainability and burnout could be alleviated by proper financial renumeration for service and engagement obligations, and for time spent writing funding applications or undertaking professional development. Economic status is an important component of social identity, while development opportunities and feeling respected are part of professional identity. Examples of unpaid labour included developing relationships with industry partners, making submissions to policymakers (e.g. Royal Commission) and teaching duties for EMCAs on contracts. These activities were expected by the University yet not remunerated, leaving EMCAs feeling undervalued.

*Promoting links with industry and opportunities to pursue that relationship with network building that is part of the work that everyone does, but is not part of the paid work that everyone does a lot of the time, particularly if you’re early career and still building your, I guess, professional identity. (Focus Group 2, P007)*

*[Unpaid teaching] is a really big X on the Faculty. They offered teaching positions to people. People put their names forward and then literally days before it was about to commence they turned around and said, we're not paying you, irrespective of your time fraction. (Focus Group 2, P008)*

EMCAs wanted to feel that all of the work they do is valued, and suggested that longer contracts, protected development time, and more internal funding opportunities would support their development and enhance financial security. Unfortunately, not all supervisors supported the development of academics in their teams:

*A standard amount of funding [should be put aside] or it's in the contract, because not all – like, I had a supervisor that just did not care at all about professional development. In actual fact, would not give you the time for it or would not give you the money for it. So standard funding and standard agreement in the contract [for grant writing and professional development]. (Focus Group 2, P008)*

#### Theme 5: Improved transitions and pathways

EMCAs identified that greater options for smooth transitions between academic opportunities across departments, as well as joint appointments, could be helpful for ensuring greater job security. Perceived role in the workplace is an important aspect of professional identity. It was expressed that there needed to be greater flexibility to move between different teams’ projects or, indeed, between research and teaching, or potential professional roles within the University.

One EMCA reflected that researchers who are trying to transition out of the research ‘gig economy’ could be placed in professional roles within the University:

*I know a lot of people who are 100 per cent researchers who want a – would like to transition out of research, but it's scary and difficult to make the jump. So I know there's some opportunities for instance at – within the business development office at the [University]. They require six-month casual employment from someone within the University. So that would be a great opportunity for a researcher, for instance, to get that work experience within the University. So make these opportunities available for them. (Focus Group 2, P009)*

Other kinds of opportunities could be made for EMCAs to increase their job security such as becoming specialised project managers for various research projects:

*So whether it's building a specialisation in project management rather than going for a specialisation in a particular topic area, things like that that are maybe not as traditionally thought of as academic skills, but if you've been on projects and managed projects before, potentially if there's a stopgap period where you don't have funding you can come on as a project manager, even though it's not as a topic specialist. (Focus Group 2, P007)*

Joint appointments between departments, health services and institutes, or with industry were also considered ideas for supporting job security:

*I know that there's a health service or I know that there's another institute that may want [an EMCA] and they can [offer a role] – so I think it's about creating some competitiveness within the – for yourself around – it's increasing your value, I think, and for that to be allowed that you can have these joint appointments and all of the obvious benefits that would come from that. (Focus Group 2, P006)*

Ultimately, EMCAs believed that increased opportunities to achieve diverse career pathways would be helpful for ensuring greater job security and satisfaction:

*There needs to be better access to diverse career pathways within the University. Often this can make a huge difference between someone completely running out of funding and leaving academia to getting that extra support for finding the next grant. (Focus Group 2, P009)*

## Discussion

Early- and mid-career academics leaving academia is a longstanding problem, but to date has received limited attention from a diversity and inclusion perspective in the medicine, dentistry and health sciences fields. This mixed-methods study sought to answer two questions, (1) What is awareness of, and engagement with, previous University- and Faculty-level diversity and inclusion initiatives? and (2) What are the opportunities and solutions to previously reported wellbeing challenges experienced by EMCAs in the workplace?

Firstly, regarding awareness and engagement, our survey identified that early- and mid-career academics may deploy workload management strategies including screening/filtering of e-mails and other advertising regarding diversity and inclusion events. Whilst there was high interest in these events, there was low awareness, and low levels of engagement, due to perceived consequences upon workload, or feeling unsupported to engage in activities outside of contractual obligations. Interest, awareness, and engagement were higher when linked with specific in-person events such as Reconciliation week, and Gender and Equality week, or the initiative addressed the desire for visible role models as highlighted in our focus groups, such as the Supporting Women in MDHS Faculty interview series. Events which had a single e-mail or newsletter notification were less known.

As previously reported, EMCAs in the workplace may experience a range of wellbeing issues including unsustainable workloads, job insecurity, power imbalances and variable supervision and mentoring support (Singh et al., 2020). Our second research question explored opportunities and solutions to these workplace challenges, and from our focus groups we constructed five themes:

1. enhanced relational support for career progression,
2. clear and transparent processes for efficient working,
3. reducing structural barriers to create opportunity,
4. improved financial renumeration, and
5. improved transitions and pathways.

The valuable contribution of work to positive mental health is highlighted in the World Health Organization’s mental health definition as “*a state of well-being in which the individual realizes his or her own abilities, can cope with the normal stresses of life, can work productively and fruitfully, and is able to make a contribution to his or her community*” [15]. Models of wellbeing encompass a range of areas linked to the workplace, such as positive relationships and emotions, purpose in life, personal growth, autonomy, engagement, accomplishment, self-acceptance and meaning (Hone et al., 2014). Workplace interventions focusing on supporting relationships, communication, learning and development, work–life balance, and quality work, are therefore proposed as disruptors of pathways to illbeing for some health professionals [16]. The work-life balance challenges for medicine, dentistry and health sciences academics identified in our study highlighted an opportunity for systems-level change to disrupt such pathways to illbeing, through initiatives that foster engagement with the University community and a sense of belonging. Partnerships with health services and medical research institutes may create unique opportunities for diverse career pathways through joint appointments that acknowledge the unique support needs of clinicians in academic roles without a clinical component [4].

The five themes related to opportunities and solutions constructed in our current study share some similarities to these disruptors of the pathway to illbeing. For example, supporting ‘relationships’ and ‘learning and development’ were reflected in the current study’s themes ‘enhanced relational support for career progression’ and ‘clear and transparent processes for efficient working’. Further, ‘work-life balance’ is reflected in the current study’s theme ‘reducing structural barriers to create opportunity’. These similarities highlight the inextricable links between wellbeing, illbeing, diversity and inclusion for early and mid-career academics, and therefore the significance of our current findings.

The unprecedented impacts of COVID-19 on the University workforce, particularly onboarding, workload, and socialisation, has likely exacerbated longstanding problems with relation to diversity and inclusion [3, 17–20]. Engagement with diversity and inclusion events could be considered a potential workplace intervention to support professional identity, celebrate social identity, promote wellbeing and prevent illbeing through fostering a sense of belonging. As such, our current findings, which suggest that EMCAs feel unable or unsupported to engage with such initiatives without, for example, direct consequences for their workload, is problematic.

This could be addressed through implementation of some of these solutions suggested by our focus group participants, such as protected development time, remuneration for other activities undertaken to support career development, and appropriate workload management such as backfill. Some countries have already committed to protecting development time for researchers, for example in the UK, a concordat enshrines 10 days of development time a year [21]. Such systems-level change has been called for in other research involving a broader population of early career academics [22], supporting our findings. Whilst there has been considerable attention towards interventions at an individual level, self-care is proposed as more sustainable through a systems approach, leveraging relationships within and with the university, as a system [23]. However, our survey findings indicate that some EMCAs perceive University-level diversity and inclusion events/initiatives as lacking systems level involvement/support, appearing superficial (“window-dressing”) or driven by the minority groups whom they concern. Strengthening systems to support a flourishing workplace through positive relationships, support and organisational culture is a yet unrealised opportunity in many institutions [19, 24–28].

In a recent rapid review of the experiences and perceptions of diversity and inclusion by early and mid-career academics employed in medicine, dentistry and health sciences, few studies were found reporting social identity and inclusion, and sexual orientation and disability were largely absent from the literature [6]. What the review did identify was evidence of job insecurity, limited advancement opportunities, and a sense of being undervalued at work. These findings were reinforced and extended within the themes identified in our current study, for example, ‘a sense of being undervalued at work’ was evident within the current study’s theme ‘improved financial renumeration’. Given this strengthened understanding, it is increasingly evident that job insecurity, limited advancement opportunities, and a sense of being undervalued at work are important factors to address in efforts to attract, sustain and retain early and mid-career academics. Our current study has identified that the experiences of these challenges are closely linked to social and professional identity, emphasising the importance of considering these issues through a diversity and inclusion lens. This could inform future theoretical modelling of academic workforce wellbeing.

### Strengths and limitations

Generalisability and transferability of our research findings is limited, despite our use of reflexivity and a process of peer-review and debriefing. We experienced limited participation of identity-diverse groups, or limited disclosure of diversity, and most of our survey respondents and all our focus group participants were women. Although a recent national survey of EMCAs (*n* = 660) comprised 71% female respondents [29], suggesting our present sample may be broadly representative, we cannot claim to evidence representation of diverse individuals in our study. This absence of some diverse groups in research was also a finding in a recent review of diversity and inclusion in the health sciences disciplines [6] and may speak to the broader barriers to identity disclosure [30].

We experienced significant difficulties with recruiting to the focus groups, and coordinating availabilities of the research participants and the research team, who were leading or participating in this project on top of their usual workload for limited remuneration. It was not possible to explore reasons for non-participation in further depth, thus we can only speculate as to why no men volunteered to participate in the focus groups. For example, the plain language statement for the project featured the full names of the research team, indicating an all-women team, which may have affected decisions by men to participate, or it could be related to women being more likely to participate in academic service activities [31, 32]. As we were unable to complete all of our planned focus groups, we may not have captured all aspects of these complex topics. Further, the focus groups being all women participants and facilitators may have impacted the content of certain themes, so findings should be interpreted with these aspects in mind. Irrespective of these limitations, this study provides an important foundation for future work focusing on strengthening organizational strategic priorities in diversity and inclusion to promote a sustainable future health sciences academic workforce.

## Conclusions

Early- and mid-career academics expressed feelings of high workload and were often unable to engage with activities outside of their immediate work responsibilities, such as events about diversity and inclusion. Implementation of key systems-based solutions to address these issues and support career progression and job security could support EMCAs with their workloads and facilitate their awareness of and ability to participate in diversity and inclusion activities and initiatives across the University and within their Faculty.

## Supplementary Information

Supplementary Material 1.

## Data Availability

The datasets used and/or analysed during the current study are available from the corresponding author on reasonable request.

## Data availability

Data is provided within the manuscript or supplementary information files. An anonymised SPSS dataset is available on request.
