# Supplementary material for: Exploring early- and mid-career academic work wellbeing challenges through a diversity and inclusion lens
Source: BMC Med Educ. 2024 Sep 27;24:1048. doi: 10.1186/s12909-024-05967-1 (PMC11428564; doi:10.1186/s12909-024-05967-1)
Supplement: Supplementary file 1 — Supplementary Material 1. [file 12909_2024_5967_MOESM1_ESM.docx]

Supplementary Materials

# Supplementary File 1. Diversity and inclusion event/initiative survey

What is the nature of your position(s) within the Faculty?

- Teaching and research (1)
- Research only (2)
- Teaching only (3)
- Academic specialist (4)
- Postgraduate research student (5)

What is the level of your appointment within the Faculty?

- Level A (1)
- Level B (2)
- Level C (3)
- Level D (4)
- Level E (5)

End of Block: Eligibility Screening

Start of Block: Part One: Demographics

Please tell us your age

- My age is (in years) (1) __________________________________________________

3. Gender: How do you identify?

- Man (1)
- Woman (2)
- Non-binary (3)
- Prefer to self-describe: (4) __________________________________________________

Please let us know if any of the following statements apply to you:

- I consider myself to be LGBTQIA+ (1)
- I am living with a disability (2)
- I am neurodiverse (3)
- I am a person of colour (4)
- I parent school age or younger children (5)
- I have caring responsibilities that are not parenting (6)
- I strongly adhere to a religious faith (7)

5. What’s your main departmental affiliation within the Faculty ? [We won’t report survey data at School/Institute level]

- Medical School (1)
- Dental School (2)
- School of Health Sciences (3)
- School of Population and Global Health (4)
- School of Psychological Sciences (5)
- School of Biomedical Sciences (6)
- Affiliated Institute, Centre or Department (7)

How long have you worked within the Faculty?

- How long I have worked within Faculty (to nearest year) (1) __________________________________________________

8. Do you work in the position(s) part time or full time?

- Full time (1)
- Part time (2)

Do you combine clinical work with your position(s)?

- Yes (please say approx how many hours a week) (1) __________________________________________________
- No (2)

What type of employment contract do you currently hold within the Faculty of Medicine, Dentistry and Health Sciences?

- Casual/Sessional (1)
- Fixed Term (less than 1 year) (2)
- Fixed Term (1-2 years) (3)
- Fixed Term (more than 2 years) (4)
- Continuous (5)

How many casual or fixed term contract renewals have you had in the time that you have worked within the Faculty of Medicine, Dentistry and Health Sciences?

- Number of contract renewals (1) __________________________________________________

Have you been awarded a PhD?

- Yes (Please tell us the year of PhD award) (1) __________________________________________________
- No but I am undertaking a PhD (2)
- No (3)

Have you, at any stage during your time within the Faculty, been awarded **external** research funding as an investigator?

- Yes (1)
- No (2)

Display This Question:

If Have you, at any stage during your time within the Faculty, been awarded external research fundin... = Yes

Have you acted as a principal investigator for any funds you’ve held?

- Yes (1)
- No (2)

Display This Question:

If Have you, at any stage during your time within the Faculty, been awarded external research fundin... = Yes

What is the largest amount of funding awarded?

- Largest amount of funding (1) __________________________________________________

Have you, at any stage during your time within the Faculty, been awarded **internal** research funding as an investigator?

- Yes (1)
- No (2)

Display This Question:

If Have you, at any stage during your time within the Faculty, been awarded internal research fundin... = Yes

Have you acted as a principal investigator for any funds you’ve held?

- Yes (1)
- No (2)

Display This Question:

If Have you, at any stage during your time within the Faculty, been awarded internal research fundin... = Yes

What is the largest amount of funding awarded?

- Largest amount of funding (1) __________________________________________________

Have you, at any stage during your time within the Faculty, been encouraged or invited to apply for a promotion, or for a post at a higher grade?

- Yes (1)
- No (2)

Would you be interested in taking part in a follow-up focus group exercise, run by early and mid career academics?
 *The focus groups will prioritise diversity and inclusion issues and solutions for the MDHS Faculty to address in their Diversity and Inclusion Action Plan.*

- Yes (1)
- No (2)

End of Block: Part One: Demographics

Start of Block: Part Two: Diversity and Inclusion Events/Initiatives

For each of the following **MDHS Faculty-level** Diversity and Inclusion events/initiatives in the last year, please indicate whether you were aware it had been advertised, whether you were interested in it, and whether you attended, applied or otherwise engaged with it.

|  | Were you aware it had been advertised? | | | Were you or would you have been interested in it? | | | Did you attend, apply or otherwise engage with it? | | |
| --- | --- | --- | --- | --- | --- | --- | --- | --- | --- |
|  | Yes (1) | No (2) | Don't recall (3) | Yes (1) | No (2) | N/A (3) | Yes (1) | No (2) | N/A (3) |
| MDHS Momentum Fellowships (1) |  |  |  |  |  |  |  |  |  |
| MDHS Diversity and Inclusion Grants (2) |  |  |  |  |  |  |  |  |  |
| MDHS Indigenous Fellowships (6) |  |  |  |  |  |  |  |  |  |
| Melbourne Disability Institute Seed Funding (11) |  |  |  |  |  |  |  |  |  |
| Supporting Women in MDHS (SWiM) Inspiring Stories seminar series (any) (3) |  |  |  |  |  |  |  |  |  |
| Supporting Women in MDHS (SWiM) Mentoring for Promotion program (7) |  |  |  |  |  |  |  |  |  |

For each of the following **MDHS Faculty-level** Diversity and Inclusion events/initiatives in the last year, please indicate whether you were aware it had been advertised, whether you were interested in it, and whether you attended, applied or otherwise engaged with it.

|  | Were you aware it had been advertised? | | | Were you or would you have been interested in it? | | | Did you attend, apply or otherwise engage with it? | | |
| --- | --- | --- | --- | --- | --- | --- | --- | --- | --- |
|  | Yes (1) | No (2) | Don't recall (3) | Yes (1) | No (2) | N/A (3) | Yes (1) | No (2) | N/A (3) |
| Innovation for Inclusion webinar (November 2021) (4) |  |  |  |  |  |  |  |  |  |
| R U OK Day webinar (September 9 2021) (5) |  |  |  |  |  |  |  |  |  |
| MDHS Diversity and Inclusion webpage 'Celebrating Diversity' stories (8) |  |  |  |  |  |  |  |  |  |
| MDHS 'Crafting a Career Interruption Statement' panel discussion video (9) |  |  |  |  |  |  |  |  |  |
| MDHS Embedding the Standards webpages (10) |  |  |  |  |  |  |  |  |  |
| Closing the gap between rhetoric and reality for people with disability panel discussion (Melbourne Disability Institute, November 2021) (11) |  |  |  |  |  |  |  |  |  |

For each of the following **University-level** Diversity and Inclusion events/initiatives in the last year, please indicate whether you were aware it had been advertised, whether you were interested in it, and whether you attended, applied or otherwise engaged with it.

|  | Were you aware it had been advertised? | | | Were you or would you have been interested in it? | | | Did you attend, apply or otherwise engage with it? | | |
| --- | --- | --- | --- | --- | --- | --- | --- | --- | --- |
|  | Yes (1) | No (2) | Don't recall (3) | Yes (1) | No (2) | N/A (3) | Yes (1) | No (2) | N/A (3) |
| Reconciliation Week (May 2021) (1) |  |  |  |  |  |  |  |  |  |
| NAIDOC week events (July 2021) (2) |  |  |  |  |  |  |  |  |  |
| Melbourne Reconciliation Network Art Forum (any) (3) |  |  |  |  |  |  |  |  |  |
| International Day of the World's Indigenous Peoples symposium (August 2021) (4) |  |  |  |  |  |  |  |  |  |
| New Disability Awareness Training (December 2021) (9) |  |  |  |  |  |  |  |  |  |
| Staff consultation for Disability Inclusion Action Plan (November 2021) (16) |  |  |  |  |  |  |  |  |  |

For each of the following **University-level** Diversity and Inclusion events/initiatives in the last year, please indicate whether you were aware it had been advertised, whether you were interested in it, and whether you attended, applied or otherwise engaged with it.

|  | Were you aware it had been advertised? | | | Were you or would you have been interested in it? | | | Did you attend, apply or otherwise engage with it? | | |
| --- | --- | --- | --- | --- | --- | --- | --- | --- | --- |
|  | Yes (1) | No (2) | Don't recall (3) | Yes (1) | No (2) | N/A (3) | Yes (1) | No (2) | N/A (3) |
| Pride in Action Ally Network: IDAHOBIT (May 2021) (5) |  |  |  |  |  |  |  |  |  |
| Pride in Action Ally Network: Midsumma activities (Pride March, Campus Lights) (May 2021) (6) |  |  |  |  |  |  |  |  |  |
| Pride in Action Ally Network: LGBTIQ+ Diversity in STEM Panel (August 2021) (7) |  |  |  |  |  |  |  |  |  |
| Pride in Action Ally Network: Training (September 2021) (8) |  |  |  |  |  |  |  |  |  |
| Gender and Sexuality at Work Conference (Melbourne Social Equity Institute, February 2022) (16) |  |  |  |  |  |  |  |  |  |

For each of the following **University-level** Diversity and Inclusion events/initiatives in the last year, please indicate whether you were aware it had been advertised, whether you were interested in it, and whether you attended, applied or otherwise engaged with it.

|  | Were you aware it had been advertised? | | | Were you or would you have been interested in it? | | | Did you attend, apply or otherwise engage with it? | | |
| --- | --- | --- | --- | --- | --- | --- | --- | --- | --- |
|  | Yes (1) | No (2) | Don't recall (3) | Yes (1) | No (2) | N/A (3) | Yes (1) | No (2) | N/A (3) |
| Gender Equality Survey (June/July 2021) (10) |  |  |  |  |  |  |  |  |  |
| Respect Taskforce Bystander Education Program (email campaign – July to September 2021) (11) |  |  |  |  |  |  |  |  |  |
| Athena Swan STEP mentoring program (December 2021) (12) |  |  |  |  |  |  |  |  |  |
| Emerging Leaders Lab: Women in STEM program (February 2022) (13) |  |  |  |  |  |  |  |  |  |
| SAGE Intersectionality Walk (March 2022) (14) |  |  |  |  |  |  |  |  |  |
| Feedback opportunity on University Gender Equality Action Plan (January 2022) (15) |  |  |  |  |  |  |  |  |  |

Is there anything else you would like to add about University-level or MDHS-level Diversity and Inclusion events/initiatives?

________________________________________________________________

________________________________________________________________

________________________________________________________________

________________________________________________________________

________________________________________________________________

End of Block: Part Two: Diversity and Inclusion Events/Initiatives

# Supplementary File 2. Focus Group Topic Guide

**Introduction**

A member of the research team will act as focus group moderator. They will welcome participants, acknowledge country, encourage introductions (name, department, role e.g. research, teaching), and explain the role participants will play in reviewing proposed solutions, generating new ones and discussing what should be prioritised.

Ground rules

• Being respectful

• Confidentiality – what is said in the room stays in the room

• Distress protocol is in place and you will be supported to seek help

• Encourage turn-taking to avoid overspeaking

**Topic presentation**

Data was presented from the 2020 MDHS EMCR survey to highlight key challenges and solutions already suggested by survey respondents. Two key challenges were presented to each group:

Group 1

- *Unsustainable workloads,*
- *Inadequate supervision,*

Group 2

- *Job insecurity,*
- *Burnout*

Participants were given 15 minutes to reflect on the proposed solutions and write out new solutions and reflections on the proposed solutions.

**Focus group discussion**

The facilitator led a discussion on all of the solutions (both generated and existing). The purpose of this discussion was to clarify, elaborate, defend or dispute the solutions, and to add any new solutions that may emerge from the discussion.
